# Crystal Field Splitting is Limiting the Stability and Strength of Ultra-incompressible Orthorhombic Transition Metal Tetraborides

**DOI:** 10.1038/srep23088

**Published:** 2016-03-15

**Authors:** R. F. Zhang, X. D. Wen, D. Legut, Z. H. Fu, S. Veprek, E. Zurek, H. K. Mao

**Affiliations:** 1 School of Materials Science and Engineering, and International Research Institute for Multidisciplinary Science, Beihang University, Beijing 100191, P. R. China; 2 Theoretical division, Los Alamos National Laboratory, Los Alamos, New Mexico 87545, USA; 3 State Key Laboratory of Coal Conversion, Institute of Coal Chemistry, Chinese Academy of Sciences, P.O. Box 165, Taiyuan, Shanxi 030001, P. R. China; 4 IT4Innovations Center, VSB-Technical University of Ostrava, CZ-708 33 Ostrava, Czech Republic; 5 Department of Chemistry, Technical University Munich, Lichtenbergstr. 4, D-85747 Garching, Germany; 6 Department of Chemistry, State University of New York at Buffalo, Buffalo, NY 14260-3000, USA; 7 Geophysical Laboratory, Carnegie Institution of Washington, NW Washington, DC 20015, USA; 8 Center for High Pressure Science and Technology Advanced Research, Shanghai 201203, P.R. China

## Abstract

The lattice stability and mechanical strengths of the supposedly superhard transition metal tetraborides (TmB_4_, Tm = Cr, Mn and Fe) evoked recently much attention from the scientific community due to the potential applications of these materials, as well as because of general scientific interests. In the present study, we show that the surprising stabilization of these compounds from a high symmetry to a low symmetry structure is accomplished by an in-plane rotation of the boron network, which maximizes the in-plane hybridization by crystal field splitting between d orbitals of Tm and p orbitals of B. Studies of mechanical and electronic properties of TmB_4_ suggest that these tetraborides cannot be intrinsically superhard. The mechanical instability is facilitated by a unique in-plane or out-of-plane weakening of the three-dimensional covalent bond network of boron along different shear deformation paths. These results shed a novel view on the origin of the stability and strength of orthorhombic TmB_4_, highlighting the importance of combinational analysis of a variety of parameters related to plastic deformation of the crystalline materials when attempting to design new ultra-incompressible, and potentially strong and hard solids.

Recently, numerous attempts to design new intrinsically ultraincompressible (bulk modulus *B* > 250 GPa) and superhard (hardness *H* ≥ 40 GPa) materials evoked much interest in the synthesis of borides of transition metals^1^,^2^,^3^,^4^,^5^,^6^ because of their potential applications, such as cutting, drilling, machining and wear-resistant tools with enhanced properties over transition metal carbides^7^,^8^,^9^. In the transition metal borides, a high density of valence electrons of transition metal (Tm) should provide such compounds with high incompressibility, and strong covalent bonds between transition metals and boron should enhance the resistance against plastic deformation. Accordingly, diborides of osmium and rhenium have been synthesized^10^,^11^. However, although they possess high elastic moduli, the correctly measured load-invariant hardness of ReB_2_ and OsB_2_ is below 30 GPa. The weak Tm-B and Tm-Tm bonds are responsible for lattice instabilities observed in these diborides^12^.

Therefore, much attention turned to the synthesis of triborides or tetraborides of transition metals in which more boron atoms are expected to form a three-dimensional (3D) covalent bond network^1^,^2^,^3^,^4^,^5^,^6^. Using the “hardness models” based on the presumption that large elastic moduli guarantee high hardness, Wang *et al.*^13^ suggested that the 5d transition metal tetraborides, such as WB_4_ and MoB_4_, should be intrinsically superhard. However, WB_4_ synthesized by Gu *et al.* has load-invariant hardness less than 30 GPa^3^, as recently confirmed by Mohammadi *et al.*^1^. The instability issue raised by Liang *et al.*^14^ and Zhang *et al.*^15^ ruled out the 3D boron network in tetraborides of 5d transition metals. Instead, triborides were proposed to be experimentally accessible because of their thermodynamic, mechanical and dynamic stability, and because of the agreement of the simulated X-ray diffraction (XRD) pattern with the experimental one^6^. In recent experiments by Chen *et al.*^16^ and Tao *et al.*^17^, the theoretically proposed structure of WB_3_ was confirmed experimentally by XRD, high-resolution transmission electron microscopy (HRTEM) and Rietveld refinement. The load-invariant hardness was about 25.5 GPa^17^, thus WB_3_ is not intrinsically superhard. The physical origin of weakening of WB_3_ lies between the metallic W-B bonds which are responsible for the lattice instability under shear deformation^6^, being similar to what has been shown in ReB_2_^18^. In view of these facts, the majority of recent attempts in the search for new superhard materials focused on transition metal borides with a 3D covalent bond network. After an exploration of several borides, researchers focused their attention on the orthorhombic TmB_4_ (Tm = Cr, Mn and Fe) in which each Tm atom is surrounded by a 3D covalent boron cage.

Several years ago, the high values of ideal shear strength of >50 GPa and hardness of ~48 GPa theoretically predicted for CrB_4_ by Niu *et al.*^19^,^20^, initiated many investigations into this material. However, the synthesized CrB_4_ has hardness only between 23.3 GPa^21^ and 28.6 GPa^22^ in agreement with the theoretical calculations by Li *et al.*^23^ Similar discrepancies between theoretical predictions and experimental results were found also for orthorhombic FeB_4_. Recently, Gou *et al.*^24^ reported a surprisingly high value of hardness for FeB_4_ of about 67 GPa for a “nanoindentation” depth of 20 to 40 nm. However, it is not clear if this value corresponds to the “asymptotic hardness” as recommended in ref. 25 for correctly measured load-invariant hardness that is obtained under conditions of fully developed plasticity^26^. This high value has been questioned by density functional theory (DFT) calculations of the anisotropic compressibility by Gou *et al.*^27^ and of the ideal strength by Li *et al.*^28^. More recently, Wang *et al.*^29^ reported a much lower hardness of synthesized FeB_4_ of 15.4 GPa in agreement with the theoretical calculations.

The question thus arises as to why the 3D covalent network in these tetraborides does not provide the presumed high hardness enhancement. For convenience, we use the nomenclature “Pearson symbol [space group number]” to denote the crystal structures, such as *oI*10[71] and *oP*10[58]. Although the *oI*10[71] structure was proposed for the experimentally synthesized CrB_4_ and FeB_4_, it has been latter found to be unstable, and should be substituted by *oP*10[58] structure^19^,^27^,^28^,^30^,^31^,^32^,^33^. The question why the low symmetry *oP*10[58] structure is energetically favorable and dynamically stable, remained so far unsolved. In order to clarify these questions, we shall study in the present work by means of DFT calculations the following problems: 1) What is the electronic origin of the stabilization from dynamically (phonon) unstable *oI*10[71] to dynamically stable *oP*10[58]? 2) What is the upper limit of mechanical strength of *oP*10[58]-TmB_4_? 3) Does the bond deformation path and electronic instability mode of TmB_4_ under shear resemble those of other borides such as ReB_2_ and WB_3_? 4) Could the three-dimensional covalent network in TmB_4_ support a higher plastic resistance?

## Results

### Stabilization from *oI*10[71]- to *oP*10[58]-TmB_4_

**Figure 1:**
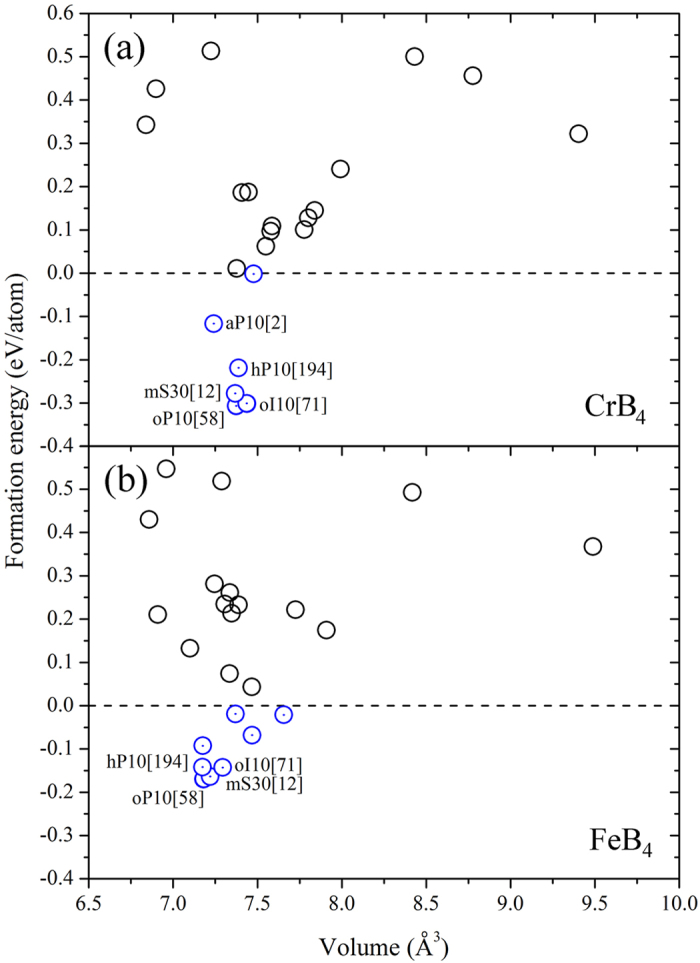
Formation energies of TmB_4_ after geometrical optimizations. Formation energy of (**a**) CrB_4_ and (**b**) FeB_4_ calculated by DFT to determine the possible ground state phases of 26 commonly observed Tm-B, Tm-Al, Tm-P, Tm-O, Tm-S ICSD structure types and the newly reported tetraborides. The most stable structure is confirmed by our evolutionary search method.

We first searched the most stable structures of stoichiometric TmB_4_ by geometry optimizations of 26 commonly observed Tm-B, Tm-C, Tm-N, Tm-Al, Tm-Si ICSD structure types^34^. The geometries were optimized using DFT as implemented in the VASP code^35^,^36^. The details of the adopted initial structures of TmB_4_ are given in the supplementary materials. The formation energy/enthalpy 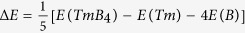 was calculated from the chemical reaction Tm + 4B = TmB_4_. The energies of α−B and Tm in magnetic state were adopted as the reference ground states. Figure 1 shows the calculated formation enthalpy of CrB_4_ and FeB_4_. The most negative formation enthalpies of oP10[58] indicate that it is the most energetically favored structure. As listed in Table S1 of the supplementary materials, eight typical structures, such as *oP*10[58], *oI*10[71], *oP*10[59], *hP*20[194], *hP*10[194], *mS*10[12], *mS*20[14] and *mS*30[12], have been reported for transition metal tetraborides. In the present study, the *oP*10[58] is found to be the most stable one for both CrB_4_ and FeB_4_. The formation enthalpies obtained in the present study for the *oI*10[71] structure of CrB_4_ (ΔH = −0.301 eV/atom) and of FeB_4_ (ΔH = −0.143 eV/atom) are slightly higher than those calculated in previous publications of ΔH = −0.3098 eV/atom for CrB_4_^37^, and of ΔH = −0.1698 eV/atom for FeB_4_^37^. Note that the initial structure with the *mS*20[14] symmetry transforms into *oP*10[58] for both CrB_4_ and FeB_4_. Our evolutionary structure search scheme^38^,^39^ confirms further that *oP*10[58] is the most stable one. A systematic thermodynamic investigation of possible ground state structures with consideration of vibrational entropy has been performed on FeB_4_ by Kolmogorov *et al.*^33^ Their results indicate that the *oP*10[58]-FeB_4_ are thermodynamically stable in the considered temperature range, but it lies 3 meV/atom above the *α*−B <−> oP12-FeB_2_ tieline at T = 0 K while 10 meV/atom below the tieline at T = 900 K^33^. The decomposition energy from FeB_4_ to *oP*12-FeB_2_ and *α*-B are calculated to be positive (~0.01 eV/atom), while the energy from FeB_4_ to *oP*8-FeB and *α*−B are negative (~ −0.02 eV/atom), in agreement with previous calculations^33^.

**Figure 2:**
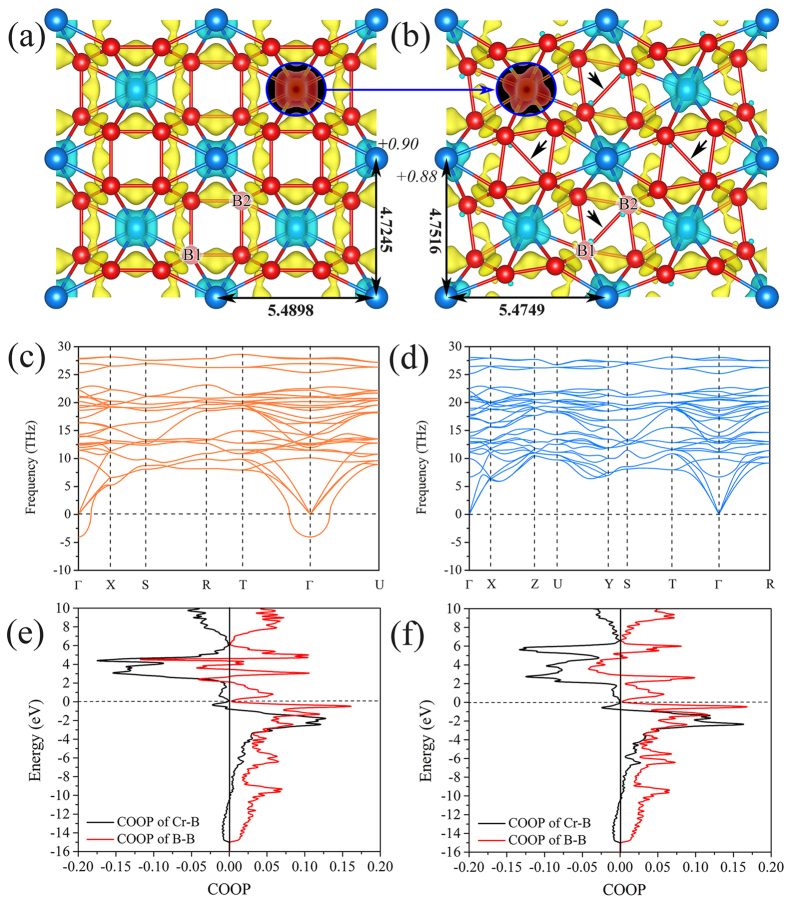
Structure and properties at equilibrium. The equilibrium bond geometry of (**a**) *oI*10[71]- and (**b**) *oP*10[58]-CrB_4_ viewed along the crystallographic [001] direction, i.e. z axis in Cartesian coordinate system such that the x and y axes are along the crystallographic [100] direction and [010] direction respectively. The isosurfaces maps of the valence charge density difference (VCDD) correspond to +/−0.01 electrons/Bohr^3^, the large blue and small red spheres represent Cr and B atoms, respectively. Calculated phonon dispersion curves for (**c**) *oI*10[71]- and (**d**) *oP*10[58]-CrB_4_. The calculated COOP curve for (**e**) *oI*10[71]- and (**f**) *oP*10[58]-CrB_4_. The numbers close to the Cr atoms in Fig. 1a,b are the corresponding Bader charges. The bold numbers close to the line arrow in (**a,b**) are the neighbor distances between two neighboring Cr-Cr atoms in unit of Angstrom.

A similar trend of thermodynamic stability is also observed for MnB_4_ in *oI*10[71] and *oP*10[58] structures. The formation enthalpy of *oI*10[71]-MnB_4_ of −0.2704 eV/atom decreases slightly by 0.01 eV/atom (0.0097 eV/atom^37^) due to the transformation to the *oP*10[58] structure whose formation enthalpy is −0.2804 eV/atom. All these calculations suggest that *oI*10[71]-TmB_4_ is thermodynamically unstable with respect to *oP*10[58]-TmB_4_. Note that a Peierls distortion mechanism has been proposed as the origin of stabilization of *mS*20[14]-MnB_4_. However, such distortion will be hindered by temperature and the phonon assisted crossover observed from nonmagnetic Peierls insulator (*mS*20) to a magnetic Stoner metal (*oP*10)^30^. Taking CrB_4_ as a prototype, Fig. 2a,b show the typical bonding feature of *oI*10[71] and *oP*10[58]-TmB_4_. Although the boron network undergoes a significant distortion during the *oI*10[71] → *oP*10[58] transformation, the molar volume changes a bit and the simulated XRD figures show a similarity between *oI*10[71] and *oP*10[58], but some minor XRD peaks show up for *oP*10[58] structure which may be identified by experiments^40^.

As the next step we calculated the phonon dispersion curves as shown in Fig. 2c,d for CrB_4_, (Figs S1 and S2 for FeB_4_ and MnB_4_). Our phonon calculations suggests that *oI*10[71] is dynamically unstable for all three tetraborides, while the *oP*10[58] structure is dynamically stable (see Fig. 2c,d for CrB_4_). Similar phonon instabilities for *oI*10[71]-FeB_4_ and *oI*10[71]-MnB_4_ are presented in the supplementary materials as Figs S1 and S2. The phonon dispersion relation of *oI*10[71]-TmB_4_ exhibits at the Γ-point imaginary phonon frequencies, thus showing its dynamic instability at T = 0 K. This result is in agreement with the previous studies of FeB_4_ by Kolmogorov *et al.*^33^ It should be noted that the *oI*10[71]-TmB_4_ structure comprised of tetragonal boron network has been previously examined within the extended Huckel method. It has been concluded that maximum binding in the 3d series is achieved for Cr, and that the electron-rich Mn, Fe, Co and Ni tetraborides may be unstable in this configuration^41^. Our present results clearly show that the dynamic instability applies for all the three *oI*10[71]-TmB_4_. In contrast, the *oP*10[58]-CrB_4_, *oP*10[58]-MnB_4_ and *oP*10[58]-FeB_4_ phases are stable as there are no imaginary modes.

With focus on the phonon-assisted transformation from *oI*10[71] to *oP*10[58], we attempted to find the electronic origin of the stabilization from higher symmetry structures to those with lower symmetry. After comparing the electronic structure of *oI*10[71]- and *oP*10[58]-CrB_4_, we found that the transformation from *oI*10[71] and *oP*10[58] induces a highly directional electronic partition at two inequivalent sites of the boron atoms in the *oP*10[58] structure: one with minor charge transfer of ~0.12 electrons, and the other one with a higher charge transfers of ~0.32 electrons. Eight equivalent boron sites are found in *oI*10[71] structure with an average Bader charge of ~0.22 electrons. The stronger anisotropy of charge transfer in *oP*10[58] structure indicates its higher electronic polarization and localization as compared to *oI*10[71] one. The Bader charge density analysis^42^ shown in Fig. 2 confirms the different charge transfer at crystallographic sites of Cr for *oI*10[71] structure (+0.90 electrons) and *oP*10[58] structure (+0.88 electrons), suggesting a slightly higher ionic contribution for the former. The anisotropy of Bader charges can be attributed to the changes from regular to distorted boron network where metal atoms are donating electrons to stabilize the *oP*10[58] structure. The analysis of crystal orbital overlap population (COOP) curves of *oI*10[71]- and *oP*10[58]-CrB_4_ shown in Fig. 2e,f, provides a further confirmation of slightly higher COOP(Cr-B) of 0.28 in *oP*10[58] structure as compared with *oI*10[71] structure (0.26). Note that an average COOP value of bonds is used to do the comparison for convenience. Similar higher values of COOP(Fe-B) (0.23) and COOP(Mn-B) (0.20) are also shown for *oP*10[58] structure as compared to *oI*10[71] structure (0.21 and 0.18 for Fe-B and Mn-B bonds respectively) (refer to Figs S3 and S4 in supplementary materials). This suggests a slightly stronger bond strength for *oP*10[58] structure. When observing the structures in Fig. 2a,b one notices much “dense” bond network in *oP*10[58] structure as compared with *oI*10[71], which is a qualitative but illustrative confirmation of the analysis of the electronic structure.

The calculated valence charge density difference (VCDD) of *oI*10[71]- and *oP*10[58]-CrB_4_ shown in Fig. 2 provide additional support of their stabilization by crystal field splitting of the d orbitals. By comparing the isosurface of VCDD of the *oI*10[71]-CrB_4_ (Fig. 2a) with that of *oP*10[58]-CrB_4_ (Fig. 2b) we see a big difference in the shape of VCDD isosurface at the metal sites: the VCDD isosurface show anisotropic shape in *oP*10[58]-CrB_4_, whereas they are relatively isotropic in *oI*10[71]-CrB_4_. This suggests that although *oP*10[58] has a lower lattice symmetry, it possess a stronger electronic directionality (see Fig. 2a,b), in agreement with the analysis of charge transfer and COOP analysis in the preceding paragraph. In order to get such electronic partition of d orbitals, the boron network in *oI*10[71] structure needs to reorganize its positions to transform into *oP*10[58] structure by rotation operations. Furthermore, the boron-boron bonds indicated by the black arrows in *oP*10[58] structure (Fig. 2b) are absent in the *oI*10[71] structure, as seen by the hollow sites in *oI*10[71] in Fig. 2a.

**Figure 3:**
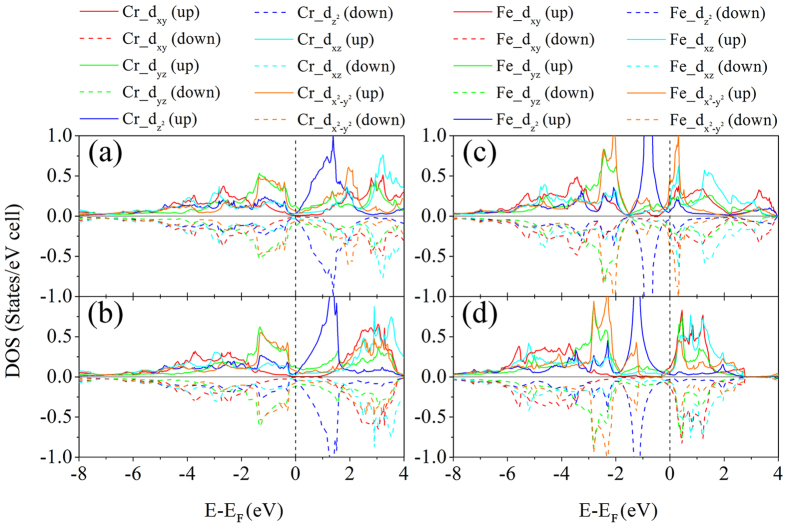
Orbital-decomposed electronic density of states of 5d orbitals of transition metals. (**a**) *oP*10[58]-CrB_4_, (**b**) *oI*10[71]-CrB_4_, (**c**) *oP*10[58]-FeB_4_ and (**d**) *oI*10[71]-FeB_4_. The transformation from *oI*10[71] to *oP*10[58] corresponds to a change of DOS shape and relative position of the decomposed d orbitals, which is more pronounced for FeB_4_. The reference Cartesian coordinates was chosen such that the x, y and z axes are along the crystallographic [100] direction, [010] direction and [001] direction respectively. The vertical dashed lines indicate the Fermi levels.

Figure 3 shows the calculated orbital decomposed electronic density of states (DOS) for CrB_4_ and FeB_4_ in *oI*10[71] and *oP*10[58] structures in their spin polarized states. Figures S5–S7 in the supplemental materials provide additional DOS curves for TmB_4_. Both structures show metallic bonding because of finite value of DOS at the Fermi level (*E*_F_), which originates mostly from the *d-*orbitals of Tm and the *p-*orbitals of B. In the *oI*10[71]-CrB_4_, the DOS around *E*_F_ (0.88 states/eV cell) is slightly higher than that in *oP*10[58]-CrB_4_ (0.80 states/eV cell), indicating a stronger “splitting” of bonding and antibonding states for *oP*10[58]-CrB_4_, in agreement with the preceding COOP analysis. A similar feature is observed for MnB_4_, but does not apply for *oI*10[71] and *oP*10[58]-FeB_4_ in which the Fermi level is located at the left shoulder of a peak in the DOS. Such differences will be shown to be responsible for their different shear moduli in the following section. Interestingly, we find in Fig. 3 a distinct change of the majority of 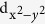 and d_xy_ orbitals (which mainly contribute to the in-plane rotation) below and above E_F_ when the structure changes from *oI*10[71] to *oP*10[58] for both CrB_4_ and FeB_4_, suggesting that crystal field splitting of d orbitals is responsible for the phonon-assisted stabilization from *oI*10[71] to *oP*10[58] as discussed above. In case of CrB_4_, the first peak of d_x_^2^_−y_^2^ below the E_F_ move upward by 0.1 eV during the transformation from *oP*10[58] to *oI*10[71], while the first peak of d_xy_ below E_F_ (at −1.167 eV) nearly disappears. For FeB_4_ however, the unexpected peaks show up at the Fermi level for *oP*10[58] structure, but not for *oI*10[71] structure.

### Mechanical properties of oP10[58]-TmB_4_

We next explore the mechanical properties of the *oP*10[58]-TmB_4_ with Tm changing from Cr to Mn and to Fe. The calculated elastic constants of *oP*10[58]-TmB_4_ are listed in Table S2 of the supplemental materials and compared with the results from other publications. It is shown that our present calculations are in good agreement with the others, and all the three orthorhombic tetraborides meet the requirements of elastic stability^43^ The Voigt average bulk and shear moduli are derived from elastic constants and compared with other hard materials in Table 1^19^,^23^,^27^,^28^,^31^,^32^,^44^,^45^,^46^,^47^,^48^. It is seen that all three *oP*10[58] tetraborides are ultra-incompressible materials owing to their high bulk moduli of more than 250 GPa. A relatively high value of shear moduli is observed for *oP*10[58]-CrB_4_ (267 GPa) and *oP*10[58]-MnB_4_ (247 GPa), suggesting they are stiff. However, a much lower value found for *oP*10[58]-FeB_4_ (192 GPa), indicates that it may be less stiff than the other two tetraborides, in agreement with the aforementioned DOS analysis that suggests a stronger metallic bonding for FeB_4_.

Well established criterion for whether a solid is ductile or brittle is if dislocation embryos can be nucleated from an atomically sharp crack prior to the crack propagation^49^,^50^. If this is the case at a given temperature, the solid is considered to be intrinsically ductile^49^,^50^. This distinction of the mechanical behavior is characterized by the ratio of the shear to bulk modulus *G*/*B*, based on the consideration that *B* is a measure of the resistance to fracture and *G* the resistance to plastic deformation^49^,^51^,^52^. The critical *G/B* ratio which separates ductile and brittle materials is around 0.57, i.e. if *G/B* < 0.57 the material is ductile, and it is brittle when *G/B* > 0.57^51^,^52^. In view of the relatively high ratio of *G/B* ≈ 0.957 for *oP*10[58]-CrB_4_, 0.879 for *oP*10[58]-MnB_4_, 0.674 for *oP*10[58]-FeB_4_, we conclude that all these tetraborides are brittle^49^,^51^. Even in brittle solids, a brittle to ductile transition is possible at an elevated temperature where the nucleation of dislocation embryos becomes possible prior to cleavage propagation^50^.

**Figure 4:**
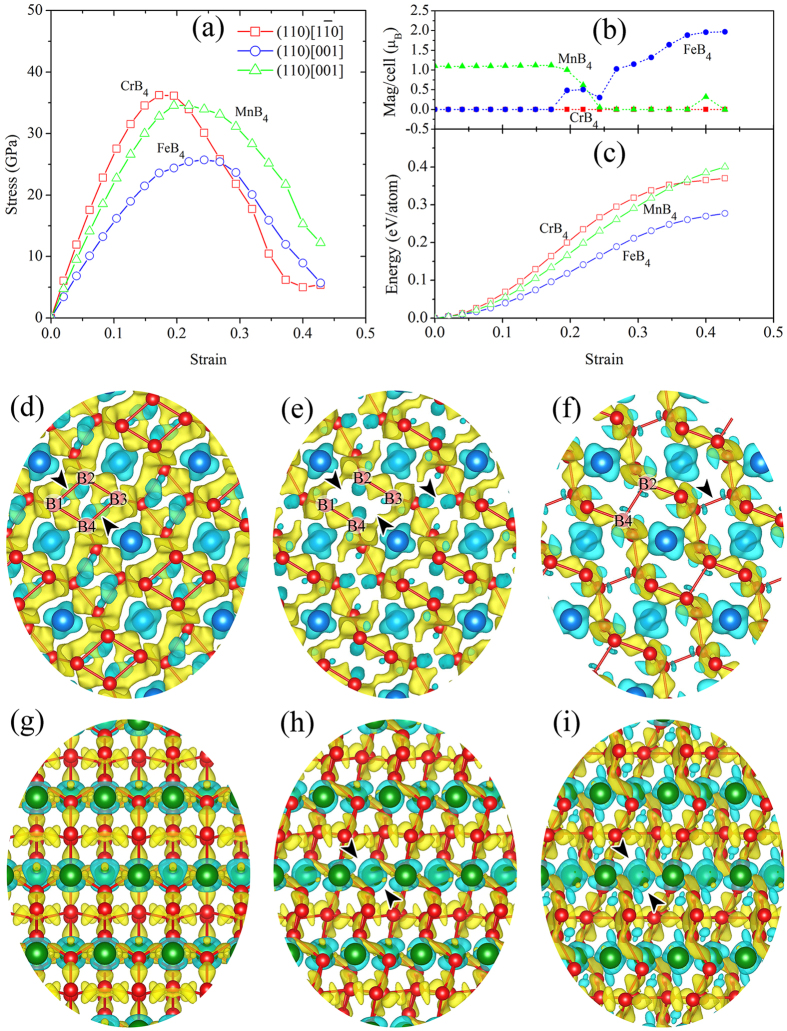
Analysis of shear deformations. (**a**) Stress-strain, (**b**) magnetic moment-strain and (**c**) energy-strain relationships for *oP*10[58]- CrB_4_, *oP*10[58]- MnB_4_ and *oP*10[58]- FeB_4_ along the weakest shearing path. The deformation paths are indicated in the figures. The snapshots of deformed bond structure and VCDD viewed along crystallographic [001] direction, i.e. z axis in Cartesian coordinate system at shear strain of (**d**) γ = 0.0000, (**e**) γ = 0.1717 (at peak) and (**f**) γ = 0.4002 (after instability) for CrB_4_ along the weakest (110)[1–10] slip system, and (**g**) γ = 0.0000, (**h**) γ = 0.2434 (at peak), and (**i**) γ = 0.4002 (after instability) for FeB_4_ along the weakest (110)[001] slip system. The black arrows in (**d–i**) indicate the position of bond instability.

It is known that high elastic moduli do not guarantee high resistance against plastic deformation because upon finite shear electronic instabilities and transformations to softer phases with lower shear resistance often occur^9^,^26^. The anisotropic ideal shear strength of *oP*10[58]-TmB_4_ may be obtained from the calculated stress-strain relationships along different crystallographic planes and directions. The slip systems of (110)[1–10] and (010)[101] were reported as the weakest ones for *oP*10[58]-CrB_4_ by Li *et al.*^23^ and by Niu *et al.*^19^, respectively. A similar disagreement exists also for the weakest slip system in FeB_4_, where (110)[001] has been reported by Li *et al.*^28^ but (111)[11–2] by Zhang *et al.*^48^. We studied all the four slip systems in more detail. Our results suggest that the (110)[1–10] slip systems is the weakest one for CrB_4_, whereas (110)[001] is the weakest for FeB_4_ and MnB_4_. Figure 4a–c show the calculated dependence of the stress, magnetic moment and total energy on strain for *oP*10[58]- CrB_4_, *oP*10[58]- MnB_4_ and *oP*10[58]- FeB_4_ along their weakest slip systems. The minimum strength for the three TmB_4_ is shown in Table 1 and compared with previous results for these tetraborides^19^,^23^,^27^,^28^,^30^,^31^,^32^, and with those of WB_3_ and MoB_3_^6^, ReB_2_^12^, OsB_2_^44^, B_6_O^45^, c-BN^46^ and diamond^45^,^47^. It is seen that the minimum shear strengths of *oP*10[58]-CrB_4_ of 36.2 GPa in the (110)[1–10] slip system are comparable to those of WB_3_ (37.7 GPa) and ReB_2_ (34.4 GPa), but they are much lower than those of c-BN (58.3 GPa^46^). This suggests that CrB_4_ cannot be intrinsically stronger than WB_3_ in view of the fact that its strength is between that of WB_3_ and ReB_2._ These results are in agreement with the recent experiments in which the load-invariant (“asymptotic”) Vickers hardness of CrB_4_ (23.3 GPa^21^ and 28.6 GPa^22^) was found to be comparable to that of ReB_2_. The high hardness of 48 GPa for CrB_4_ “predicted” by Niu *et al.*^19^ on the basis of the “hardness model” is not justified. Similar conclusions apply also for MnB_4_ and FeB_4_ because a much lower ideal shear strength is found for *oP*10[58]-MnB_4_ (35.1 GPa) and *oP*10[58]-FeB_4_ (25.7 GPa), although Niu *et al.*^19^ predicted a high value of hardness of 41.5 GPa for MnB_4_. The reason is simply the fact that the “theoretical models of hardness” compares in fact elastic stiffness, and do not account for electronic instabilities upon a finite shear^18^,^26^.

### Bond deformation mechanism of *oP*10[58]-TmB_4_

In view of the mechanical weakness of *oP*10[58]-TmB_4_, we investigated the bond deformation paths and electronic instability mode of *oP*10[58]-TmB_4_ along the weakest shear path. Figure 4a,c show that both stress-strain and total energy-strain curves are smooth, i.e. no distinct electronic instabilities occur as in the cases of ReB_2_^18^ and WB_3_^6^, but not for the change of magnetic moment for FeB_4_. Figure 4d–f show the variation of the VCDD from the equilibrium (γ = 0.0000), to a shear strain γ = 0.1717 corresponding to the stress maximum and at shear strain γ = 0.4002 after the shear instability for CrB_4_ in the weakest (110)[1–10] slip system. One can see the change of in-plane lengths of the B1–B2 and B3–B4 bonds with respect to (001) planes from ~1.85 Å at equilibrium to ~2.11 Å at maximum stress. With the elongation of B1–B2 and B3–B4 bond lengths, the magnitude of charge depletion between them increases gradually before the stress reaches the peak value of about 25.7 GPa (see Fig. 4e). After the peak stress, the B2 atom and B4 atom come closer together and form B2–B4 bonds with length of ~1.97 Å at a strain of 0.4002, while the B1 and B3 atoms further separate. The shear deformation after the peak stress is accompanied by a stress decrease to about 5 GPa (Fig. 4a).

In FeB_4_ and MnB_4_, the weakest link does not appear along the (110)[1–10] shear. Therefore we concentrate on the deformation within the (110)[001] slip system. In view of the similarity between FeB_4_ and MnB_4_, we shall take FeB_4_ as an illustrative example. Figure 4g–i show the variation of the VCDD isosurfaces in equilibrium (γ = 0.0000), at a shear strain of γ = 0.2434 corresponding to the maximum stress, and at γ = 0.4002 after the shear instability of FeB_4_, respectively. No significant bond weakening or breaking appear before the shear stress reaches the maximum value at strain of about 0.25. After the shear instability, a multiple out-of-plane bond weakening and breaking appears between the {001} planes (see the regions marked by black arrows in Fig. 4h,i). In the following orbital analysis we shall show that such bond weakening is attributed to the electronic instability between the d orbitals of Fe and p orbitals of B, which are responsible for the out-of-plane bonding between {001} planes.

### Electronic origin of lattice instability under shearing

**Figure 5:**
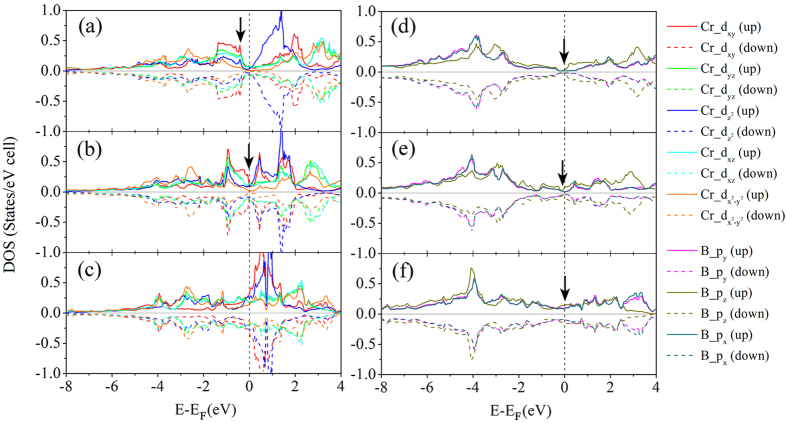
Orbital-decomposed electronic density of states of *oP*10[58]-CrB_4_. (**a**,**d**) at equilibrium, (**b**,**e**) at a strain of 0.1717, (**c**,**f**) at a strain of 0.4002. The reference Cartesian coordinates was chosen such that the x axis is normal to crystallographic (1–10) plane, the y axis is along the crystallographic [110] direction and the z axis along the [001] direction respectively. The black arrows in (**a**–**c**) indicate the change of d_xy_ state, and those in (**d**–**f**) show the change of pseudogap at Fermi level.

For that purpose, we analyze the change of orbital-decomposed DOS of d orbitals of Tm and p orbitals of boron in TmB_4_ before and after lattice instability. Figure 5a–f shows the orbital-decomposed DOS of CrB_4_ at equilibrium, at a strain of 0.1717, and at a strain of 0.4002. Upon shear but before the lattice instability, the occupied d_xy_ states located below *E*_*F*_ move towards *E*_*F*_ (see the red curve between −2 eV and 0 eV in Fig. 5a,b), while the peak of d_xy_ above *E*_*F*_ move downwards and split into two parts between 0 and 1 eV, and between 1 and 2 eV. The splitting feature is also observed for the d_z_^2^ states above *E*_*F*_ (see blue curve between 0 eV and 2 eV in Fig. 5a,b). During this process, the shape of the other three d-states around *E*_*F*_ does not change significantly. After the lattice instability (see Fig. 5c), most of the d_xy_ and d_z_^2^ states between −2 eV and 0 eV have vanished, and both contribute substantially to peaks in the DOS between 0 and 2 eV above *E*_*F*_. At the same time, the pseudogap in the p_z_ states of boron became narrower and finally vanished, while p_x_ and p_y_ increase their contribution at *E*_*F*_ after the lattice instability. At the peak stress, all three p orbitals contribute to the states at *E*_*F*_. Therefore, the change of the DOS arising from the d_xy_ orbitals of Cr and from p_x_ and p_y_ orbitals of B are mainly responsible for the in-plane bond weakening of B1–B2 and B3–B4 bonds within the x–y plane, as shown above.

**Figure 6:**
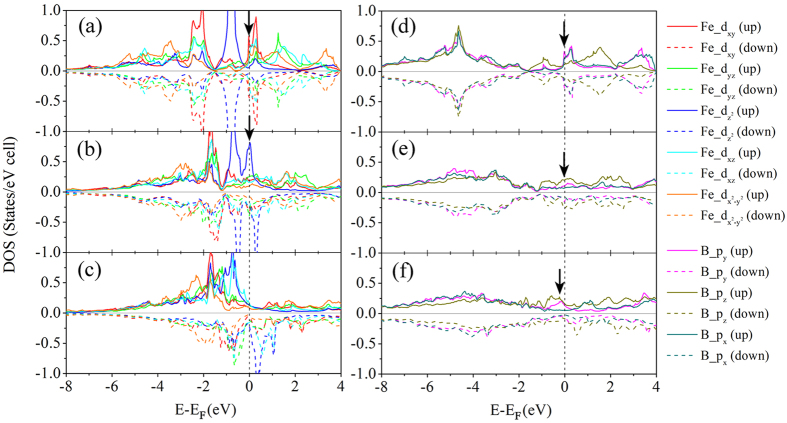
Orbital-decomposed electronic density of states of *oP*10[58]-FeB_4_. (**a**,**d**) at equilibrium, (**b**,**e**) at a strain of 0.2434, (**c**,**f**) at a strain of 0.4002. The reference Cartesian coordinate was chosen such that the x axis is normal to crystallographic (1–10) plane, the y axis is along the crystallographic [110] direction and the z axis along [001] direction respectively. The black arrows in Fig. (**a**–**c**) indicate that the major contributions at Fermi level changes from d_xy_ to d_z_^2^ state during shear, while arrows shown in Fig. (**d**–**f**) show the change of p_z_ state at Fermi level.

Figure 6 shows the orbital-decomposed EDOS of the (110)[001] slip system of FeB_4_ in equilibrium, at a strain of 0.2434 corresponding to the stress maximum, and at strain 0.4002 after the shear instability. In equilibrium, dz^2^ states show a pseudogap at *E*_*F*_ whereas the other four d orbitals contribute mostly to the finite value at *E*_*F*_. Before the instability, the peaks of all five orbitals between −6 eV and 0 eV move towards *E*_*F*_, and at the maximum stress one profound peak of d_z_^2^ character is seen at the Fermi level giving large contribution to the states at *E*_*F*_, while nearly all peaks of other spin-up orbitals above the Fermi level vanish. After the lattice instability, the states of all spin-down orbitals move further upwards, whereas the d_xz_ spin-down states appear above Fermi level. The appearance of boron p_z_ states at the Fermi level and the decrease of peaks of p_x_ and p_y_ states can be clearly seen in Fig. 6d–f. Therefore we can conclude that the electronic instability of FeB_4_ during (110)[001] shear is mainly due to the changes of the energies of states of Fe d_z_^2^ and d_xz_ orbitals, and of p_z_ orbitals of B. MnB_4_ shows a similar instability as FeB_4_, therefore we do not discuss it here in any further detail (see Fig. S8 in supplemental materials).

The electronic instability of TmB_4_ (Tm = Cr, Mn and Fe) upon shear due to d orbitals of Tm and p orbitals of B does not resemble the cases of ReB_2_^18^ and WB_3_^6^. The important difference is that in TmB_4_ the covalent bond networks are mainly responsible for the shear instability, while metal-boron or metal-metal bonds are the carrier of the shear instability in ReB_2_ and WB_2_. Although the three dimensional covalent network has been proposed to be responsible for high hardness in transition metal borides for a long time, our results show uniquely that the 3D boron network in the tetraborides may not be strong enough to provide these materials with high plastic resistance. Because the strength of a real crystalline material is limited by a variety of defects, such as dislocations, cracks, grain boundaries and others, it is usually orders of magnitude smaller than that of an ideal crystal. The Peierls–Nabarro (PN) stress of dislocations provides a realistic explanation on plastic resistance of a real crystal and can be correlated to shear moduli and ideal shear strength^53^,^54^,^55^ as 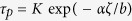, where α = 2π and *K* = *G*/(1−ν) for edge dislocation and *K* = ***G*** for screw dislocation, *b* is the Burgers vector, ν is the Poisson ratio, and ζ is the dislocation half-width which can be expressed as 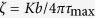, where *τ*_max_is the ideal shear strength. Thus, the plastic resistance of a crystal depends mainly on the shear modulus, ideal shear strengths, lattice topology, bonding nature and deformation mode. The present work emphasizes the necessity to conduct a combinational analysis of the lattice stability, of the values of shear moduli, of the anisotropic shear strength, of the complexity of lattice topology and its changes during shear, of the nature of chemical bonding, and others which are critical parameters in Peierls–Nabarro dislocation model of plastic lattice resistance^53^,^54^,^55^. Despite a century of research on dislocation mediated plasticity, a systematic connection to those quantities derived from first principle calculations has not been rationalized, inducing massive applications of a single parameter e.g. elastic moduli or ideal shear strength, to directly quantify the mechanical strength and hardness of a real material^3^,^4^,^9^,^10^,^11^,^12^,^19^,^23^,^24^,^28^.

## Discussion

In summary, we carried out comprehensive density functional theory calculations to determine the thermodynamic, mechanical and phonon stabilities, the deformation paths and the electronic instability modes of orthorhombic TmB_4_. The electronic structure calculations reveal that the transformation from *oI*10[71]-TmB_4_ to *oP*10[58]-TmB_4_ can be explained by the variation of the in-plane electronic structure within (001) planes and the formation of new boron-boron bonds at hollow sites by distortion of boron networks. These processes significantly enhance the electronic hybridization of d orbitals of Tm with p orbitals of B by crystal field splitting. Depending on the different deformation paths (slip systems), different orbitals are responsible for the electronic instability upon finite shear. The relatively low shear moduli and ideal strengths of TmB_4_ suggest that these materials cannot be intrinsically superhard. The instability of weak 3D boron covalent networks is found to be responsible for the weakness of *oP*10[58]-TmB_4_. These results illustrate the importance and necessity of a combinational analysis of a variety of parameters related to plastic deformation of the crystalline materials, and their combination in the attempt to design new intrinsically hard and superhard materials.

## Methods

### First principles calculations

The present first-principles density functional theory (DFT) calculations of the formation energy of TmB_4_, Tm = Cr, Mn and Fe were done by means of the VASP code^35^ with the projector augmented wave method^36^ employed to describe the electron-ion interaction and the generalized-gradient approximation of Perdew-Burke-Ernzerhof (PBE)^56^ for the exchange correlation term. The integration in the Brillouin zone has been done on special *k* grids for the phases that were under consideration, which were determined according to the Monkhorst-Pack scheme, the energy cutoff of 600 eV, and the tetrahedron method with Blöchl corrections for the density of states and smearing methods for the stress calculations. The verification of the reliability of our calculations has been done by calculating the total energies, equilibrium lattice parameter, and bulk modulus of TmB_4_ and compared with previous theoretical and experimental values in the present work, and of a number of other materials in our earlier work. In the present studies, all DFT calculations are performed with spin-polarized scheme because of the appearance of magnetic momentum during the deformation. Nevertheless, in cases where no magnetic momentum has been found, additional non-spin-polarized calculation is used to check its possible influence.

### The phonon dispersion

The phonon calculations were performed within the harmonic approximation using the direct method^57^ based on the calculated non-vanishing Hellman-Feynman forces employing the Phonopy code^58^. To confirm our results, we also used a linear response method based on the perturbation theory as implemented in VASP code.

### The elastic constants

For orthorhombic crystals (Group numbers 16–74), there are nine independent elastic constants usually referred to as *c*_11_, *c*_22_ , *c*_33_ , *c*_44_ , *c*_55_ , *c*_66_ , *c*_12_ , *c*_13_ , and *c*_23_. In the following, we give a set of simple strain configurations and the corresponding strain-energy density variations Δ*E*/*V*_0_. (r1) *ε* = (*δ,*0,0,0,0,0) with relation 
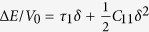
, (r2) *ε* = (0,*δ*,0,0,0,0) with relation 
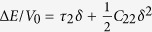
, (r3) *ε* = (0,0,*δ*,0,0,0) with relation 
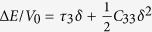
, (r4)*ε* = (0,0,0,*δ*,0,0) with relation 
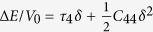
, (r5)*ε* = (0,0,0,0,*δ,0*) with relation 
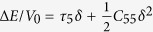
, (r6)*ε* = (0,0,0,0,0,*δ*) with relation 
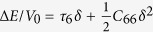
, (r7)*ε* = (*δ*,*δ*,0,0,0,0) with relation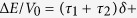 
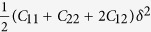
, (r8)*ε* = (*δ*,0,*δ*,0,0,0) with relation 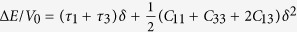, (r9)*ε* = (0,*δ*,*δ*,0,0,0) with relation 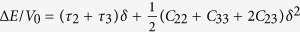.

### The stress-strain relationships

First, the atomic basis vectors of a given crystal cell were projected onto the Cartesian coordinates *R* with one axis vector being parallel to the imposed strain direction for the tension. For the shear, one axis vector was perpendicular to the slip plane and another one was parallel to the slip direction in that plane. Afterwards, the crystal has been incrementally deformed by transforming the unstrained atomic basis vector matrices *R* to the strained ones *R*′ using the deformation matrices.


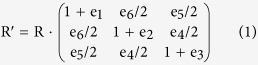


where *e*_1_ = *e*_*xx*_, *e*_2_ = *e*_*yy*_, *e*_3_ = *e*_*zz*_, *e*_4_ = *e*_*zy*_ + *e*_*yz*_, *e*_5_ = *e*_*zx*_ + *e*_*xz*_, and *e*_6_ = *e*_*yx*_ + *e*_*xy*_ are the strain components in the Voigt notation. The diagonal strain components *e*_*xx*_, *e*_*yy*_, and *e*_*zz*_ represent the tension while the off-diagonal components represent the shear. In order to keep the crystal under a stress state of uniaxial tension or shear, the strained cell has been relaxed for both the atomic basis vectors and for the atom coordinates inside the unit cell by keeping the applied strain component fixed and relaxing the other five strain components until their conjugate stress components i.e., Hellmann–Feynman stresses reached negligible values. Such a relaxation scheme is accomplished by using a slightly modified VASP code with specific constraints of strain components. To ensure that the strain path is continuous, the starting position at each strain step has been taken from the relaxed coordinates of the previous strain step. In the instance of having a large strain, the crystal symmetry may be changed and the Brillouin zone significantly deformed. Therefore we adopt a high energy cutoff of 600 eV and verified the convergence of the calculations of the stress-strain curves by using different meshes of *k* points. Although the spin-polarized calculation does not have big impact on the shear strength on FeB_4_ and CrB_4_, we performed the spin-polarized calculations for a comparison because of the significant magnetic effect on the results of MnB_4_ and possible appearance of magnetic moment during severe deformation.

### Evolutionary Structure Searches

Structure searches were performed using the open-source evolutionary algorithm (EA) XtalOpt^38^,^39^ along with the default parameter set from ref. 38. EA runs were carried out on the TmB_4_ with 2, 3 and 4 formula units in the primitive cell. In this algorithm a new offspring is procreated as soon as an individual is optimized and the parents are chosen from a population based pool. The population size were around 735, 225, 110 for the 2, 3 and 4 formula unit cells of CrB_4_, respectively, and 200, 225, 160 for the 2, 3 and 4 formula unit cells of MnB_4_, respectively. In each run the same low enthalpy structure was found at least 3 times (often more) before the run was terminated. A six-step structural-optimization scheme was used for all runs, and each step employed the geometry of the structure from the previous step for the initial geometry. Only the ions were allowed to relax in the first two steps, while the last four steps also allowed the lattice parameters to relax. The precision of the calculation was increased at each step. The calculations were performed using the PBE exchange-correlation functional, the PAW method and an energy cut-off of 450 eV in the final step. The lowest energy structures from each search were optimized using the aforementioned computational settings, to obtain a more accurate energy ranking.

## Additional Information

**How to cite this article**: Zhang, R. F. *et al.* Crystal Field Splitting is Limiting the Stability and Strength of Ultra-incompressible Orthorhombic Transition Metal Tetraborides. *Sci. Rep.* **6**, 23088; doi: 10.1038/srep23088 (2016).

## Supplementary Material

Supplementary Information

## Figures and Tables

**Table 1:** Voigt bulk modulus *B*_*V*_, shear modulus *G*_*V*_, and ideal strength (minimum tensile strength ***σ***_min_ and shear strength ***τ***_min_) of transition metal tetraborides (TmB_4_, Tm = Cr, Mn, Fe) calculated by first principles methods.

Compound	Reference	*B*_*V*_	*G*_*V*_	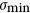	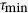
CrB_4_	This study	279	267	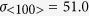	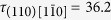
	19	265	261	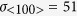	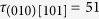
	23	263	267		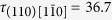
	31	237*	255*		
MnB_4_	This study	281	247	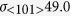	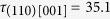
	19	270	245		
FeB_4_	This study	285	192	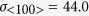	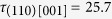
	19	253	177		
	28	284	194		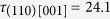
	48	265	198	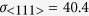	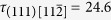
	27	268	189		
	31	250*	181*		
	32	277	186		
WB_3_	6	293	245	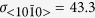	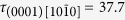
MoB_3_	6	276	226	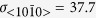	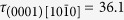
OsB_2_	44	313	181	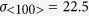	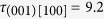
ReB_2_	12	348	274	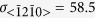	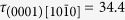
B_6_O	45	231	218	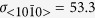	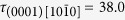
BN	46	376	390	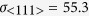	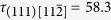
Diamond	45,47	442	528	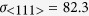	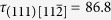

Previous theoretical results for WB_3_^6^, MoB_3_^6^, OsB_2_^44^, ReB_2_^12^, B_6_O^45^, BN^46^ and Diamond^45^,^47^ are included for comparison. *is calculated using Voigt-Reuss-Hill approximation

## References

[b1] Mohammadi R. *et al.* an inexpensive superhard material. P. Natl. Acad. Sci. USA 108, 10958–10962 (2011). 10.1073/pnas.1102636108 PMC3131357 21690363

[b2] Mohammadi R. *et al.* Toward inexpensive superhard materials: tungsten tetraboride-based solid solutions. J. Am. Chem. Soc. 134, 20660–20668 (2012). 23171079 10.1021/ja308219r

[b3] Gu Q., Krauss G. & Steurer W. Transition Metal Borides: Superhard versus ultra-incompressible. Adv. Mater. 20, 3620 (2008).

[b4] Levine J. B., Tolbert S. H. & Kaner R. B. Advancements in the search for superhard ultra-incompressible metal borides. Adv. Funct. Mater. 19, 3519–3533 (2009).

[b5] Li Q., Zhou D., Zheng W. T., Ma Y. M. & Chen C. F. Global structural optimization of tungsten borides. Phys. Rev. Lett. 110, 136403 (2013). 23581349 10.1103/PhysRevLett.110.136403

[b6] Zhang R. F. *et al.* Stability and strength of transition-metal tetraborides and triborides. Phys. Rev. Lett. 108, 255502 (2012). 23004618 10.1103/PhysRevLett.108.255502

[b7] Lech A. T., Turner C. L., Mohammadi R., Tolbert S. H. & Kaner R. B. Structure of superhard tungsten tetraboride: A missing link between MB_2_ and MB_12_ higher borides. P. Natl. Acad. Sci. USA 112, 3223–3228 (2015). 10.1073/pnas.1415018112 PMC4371990 25733870

[b8] Kotmool K. *et al.* Revealing an unusual transparent phase of superhard iron tetraboride under high pressure. P. Natl. Acad. Sci. USA 111, 17050–17053 (2014). 10.1073/pnas.1419244111 PMC4260608 25404295

[b9] Veprek S. Recent search for new superhard materials: Go nano! J. Vac. Sci. Technol. A 31, 050822 (2013).

[b10] Cumberland R. W. *et al.* Osmium diboride, an ultra-incompressible, hard material. J. Am. Chem. Soc. 127, 7264–7265 (2005). 15898746 10.1021/ja043806y

[b11] Chung H. Y. *et al.* Synthesis of ultra-incompressible superhard rhenium diboride at ambient pressure. Science 316, 436–439 (2007). 17446399 10.1126/science.1139322

[b12] Zhang R. F., Veprek S. & Argon A. S. Mechanical and electronic properties of hard rhenium diboride of low elastic compressibility studied by first-principles calculation. Appl. Phys. Lett. 91, 201914 (2007).

[b13] Wang M., Li Y. W., Cui T., Ma Y. M. & Zou G. T. Origin of hardness in WB_4_ and its implications for ReB_4_, TaB_4_, MoB_4_, TcB_4_, and OsB_4_. Appl. Phys. Lett. 93, 101905 (2008).

[b14] Liang Y. C., Yuan X. & Zhang W. Q. Thermodynamic identification of tungsten borides. Phys. Rev. B 83, 220102 (2011).

[b15] Zhang M. G., Wang H., Wang H. B., Cui T. & Ma Y. M. Structural modifications and mechanical properties of molybdenum borides from first principles. J. Phys. Chem. C 114, 6722–6725 (2010).

[b16] Cheng X. *et al.* Interstitial-boron solution strengthened WB_3 + *x*_. Appl. Phys. Lett. 103, 171903 (2013).

[b17] Tao Q. *et al.* Exploring hardness and the distorted sp^2^ hybridization of B-B Bonds in WB_3_. Chem. Mater. 26, 5297–5302 (2014).

[b18] Zhang R. F., Legut D., Niewa R., Argon A. S. & Veprek S. Shear-induced structural transformation and plasticity in ultraincompressible ReB_2_ limit its hardness. Phys. Rev. B 82, 104104 (2010).

[b19] Niu H. Y. *et al.* Structure, bonding, and possible superhardness of CrB_4_. Phys. Rev. B 85, 144116 (2012).

[b20] Niu H. Y. *et al.* Variable-composition structural optimization and experimental verification of MnB_3_ and MnB_4_. Phys. Chem. Chem. Phys. 16, 15866–15873 (2014). 24962459 10.1039/c4cp01339e

[b21] Knappschneider A. *et al.* Possible superhardness of CrB_4_. Inorg. Chem. 52, 540–542 (2013). 23298445 10.1021/ic3020404

[b22] Wang S. *et al.* Crystal structures, elastic properties, and hardness of high-pressure synthesized CrB_2_ and CrB_4_. J. Superhard Mater. 36, 279–287 (2014).

[b23] Li B., Sun H., Zang C. P. & Chen C. F. Fundamental constraints on the strength of transition-metal borides: The case of CrB_4_. Phys. Rev. B 87, 174106 (2013).

[b24] Gou H. Y. *et al.* Discovery of a superhard iron tetraboride superconductor. Phys. Rev. Lett. 111, 157002 (2013). 24160619 10.1103/PhysRevLett.111.157002

[b25] Brazhkin V. *et al.* What-does ‘harder than diamond’ mean? Nat. Mater. 3, 576–577 (2004). 15343282 10.1038/nmat1196

[b26] Veprek S., Zhang R. F., Veprek-Heijman M. G. J., Sheng S. H. & Argon A. S. Superhard nanocomposites: Origin of hardness enhancement, properties and applications. Surf. Coat. Tech. 204, 1898–1906 (2010).

[b27] Gou Y. P., Fu Z., Liang Y. C., Zhong Z. & Wang S. M. Electronic structures and mechanical properties of iron borides from first principles. Solid St. Comm. 187, 28–32 (2014).

[b28] Li B., Sun H. & Chen C. F. First-principles calculation of the indentation strength of FeB_4_. Phys. Rev. B 90, 014106 (2014).

[b29] Wang Q. Q. *et al.* Is orthorhombic iron tetraboride superhard? J. Materiomics 1, 45–51 (2015).

[b30] Liang Y. C., Yuan X., Gao Y. F., Zhang W. Q. & Zhang P. H. Phonon-assisted crossover from a nonmagnetic Peierls Insulator to a magnetic Stoner metal. Phys. Rev. Lett. 113, 176401 (2014). 25379925 10.1103/PhysRevLett.113.176401

[b31] Yang M. *et al.* Structural distortion and band gap opening of hard MnB_4_ in comparison with CrB_4_ and FeB_4_. J. Solid State Chem. 213, 52–56 (2014).

[b32] Zhang X. Y. *et al.* First principle study of elastic and thermodynamic properties of FeB_4_ under high pressure. J. Appl. Phys. 114, 183517 (2013).

[b33] Kolmogorov A. N. *et al.* New Superconducting and Semiconducting Fe-B Compounds Predicted with an Ab Initio Evolutionary Search. Phys. Rev. Lett. 105, 217003 (2010). 21231344 10.1103/PhysRevLett.105.217003

[b34] ICSD-Inorganic Crystal Structure Database, http://www2.fiz-karlsruhe.de/icsd_home.html (Date of access: 08/07/2014).

[b35] Kresse G. & Furthmüller J. Efficiency of ab-initio total energy calculations for metals and semiconductors using a plane-wave basis set. Comput. Mater. Sci. 6, 15–50 (1996). 10.1103/physrevb.54.11169 9984901

[b36] Kresse G. & Joubert J. From ultrasoft pseudopotentials to the projector augmented-wave method. Phys. Rev. B 59, 1758 (1999).

[b37] Van der Geest A. G. & Kolmogorov A. N. Stability of 41 metal-boron systems at 0 GPa and 30 GPa from first principles. Calphad 46, 184–204 (2014).

[b38] Lonie D. C. & Zurek E. XTALOPT: An open-source evolutionary algorithm for crystal structure prediction. Comput. Phys. Commun. 182, 372–387 (2011).

[b39] Lonie D. C. & Zurek E. XTALOPT version r7: An open-source evolutionary algorithm for crystal structure prediction. Comput. Phys. Commun. 182, 2305–2306 (2011).

[b40] Bialon A. F. *et al.* Possible routes for synthesis of new boron-rich Fe-B and Fe_1−x_Cr_x_B_4_ compounds. Appl. Phys. Lett. 98, 081901 (2011).

[b41] Burdett J. K. & Canadell E. Chromium boride (CrB_4_) and manganese boride (MnB_4_): electronic structures of two unusual systems containing the tetragonal carbon net. Inorg. Chem. 27, 4437–4444 (1988).

[b42] Bader R. F. W. In Atoms in molecules-A quantum theory 1 (Oxford University Press, 1990).

[b43] Wu Z. J. *et al.* Crystal structures and elastic properties of superhard IrN_2_ and IrN_3_ from first principles. Phys. Rev. B 76, 054115 (2007).

[b44] Zhang R. F. *et al.* Bond deformation paths and electronic instabilities of ultraincompressible transition metal diborides: Case study of OsB_2_ and IrB_2_. Phys. Rev. B 90, 094115 (2014).

[b45] Zhang R. F., Lin Z. J., Zhao Y. S. & Veprek S. Superhard materials with low elastic moduli: Three-dimensional covalent bonding as the origin of superhardness in B_6_O. Phys. Rev. B 83, 092101 (2011).

[b46] Zhang R. F., Veprek S. & Argon A. S. Anisotropic ideal strengths and chemical bonding of wurtzite BN in comparison to zincblende BN. Phys. Rev. B 77, 172103 (2008).

[b47] Zhang R. F., Lin Z. J. & Veprek S. Anisotropic ideal strengths of superhard monoclinic and tetragonal carbon and their electronic origin. Phys. Rev. B 83, 155452 (2011).

[b48] Zhang M. *et al.* Hardness of FeB_4_: Density functional theory investigation. J. Chem. Phys. 140, 174505 (2014). 24811644 10.1063/1.4871627

[b49] Rice J. R., Beltz G. E. & Sun Y. In Topics in fracture and fatigue (ed. Argon A. S.) 1–58 (Springer, 1992).

[b50] Xu G., Argon A. S. & Ortiz M. Critical configurations for dislocation nucleation from crack tips. Philos. Mag. A 75, 341–367 (1997).

[b51] Pugh S. F. X. C. I. I. Relations between the elastic moduli and the plastic properties of polycrystalline pure metals. Philos. Mag. A 45, 823–843 (1954).

[b52] Mattesini M., Ahuja R. & Johansson B. Cubic Hf_3_N_4_ and Zr_3_N_4_: A class of hard materials. Phys. Rev. B 68, 184108 (2003).

[b53] Zhang R. F., Sheng S. H. & Veprek S. Origin of different plastic resistance of transition metal nitrides and carbides: Stiffer yet softer. Scripta Mater. 68, 913–916 (2013).

[b54] Yu X. H. *et al.* High pressure phase-transformation induced texture evolution and strengthening in zirconium metal: experiment and modeling. Sci. Rep. 5, 12552 (2015). 26218405 10.1038/srep12552 PMC4517392

[b55] Argon A. S. In Strengthening mechanisms in crystal plasticity 78 (Oxford University Press, 2008).

[b56] Perdew J. P., Burke K. & Ernzerhof M. Generalized gradient approximation made simple. Phys. Rev. Lett. 77, 3865 (1996). 10062328 10.1103/PhysRevLett.77.3865

[b57] Parlinski K., Li Z. Q. & Kawazoe Y. First-principles determination of the soft mode in cubic ZrO_2_. Phys. Rev. Lett. 78, 4063 (1997).

[b58] Togo A., Oba F. & Tanaka I. First-principles calculations of the ferroelastic transition between rutile-type and CaCl_2_-type SiO_2_ at high pressures. Phys. Rev. B 78, 134106 (2008).

