# Supplementary material for: Crystal Field Splitting is Limiting the Stability and Strength of Ultra-incompressible Orthorhombic Transition Metal Tetraborides
Source: Sci Rep. 2016 Mar 15;6:23088. doi: 10.1038/srep23088 (PMC4791561; doi:10.1038/srep23088)
Supplement: Supplementary Information [file srep23088-s1.pdf]

# Supplementary Information

# Crystal Field Splitting is Limiting the Stability and Strength of Ultra-incompressible Orthorhombic Transition Metal Tetraborides

R. F. Zhang[1,2,*], X. D. Wen[2,3], D. Legut[4], Z. H. Fu[1], S. Veprek[5], E. Zurek[6] & H. K. Mao[7]

[1]*School of Materials Science and Engineering, and International Research Institute for Multidisciplinary Science, Beihang University, Beijing 100191, P. R. China*

[2]*Theoretical division, Los Alamos National Laboratory, Los Alamos, New Mexico 87545, USA*

[3]*State Key Laboratory of Coal Conversion, Institute of Coal Chemistry, Chinese Academy of Sciences, P.O. Box 165, Taiyuan, Shanxi 030001, P. R. China*

[4]*IT4Innovations Center, VSB-Technical University of Ostrava, CZ-708 33 Ostrava, Czech Republic*

[5]*Department of Chemistry, Technical University Munich, Lichtenbergstr. 4, D-85747 Garching, Germany*

[6]*Department of Chemistry, State University of New York at Buffalo, Buffalo, NY 14260-3000, USA*

[7]*Geophysical Laboratory, Carnegie Institution of Washington, NW Washington, DC 20015, USA; Center for High Pressure Science and Technology Advanced Research, Shanghai 201203, P.R. China*

**Table S1. Library of structure types for $Me_1Le_4$ compounds (Me=metals or Si; Le=B, Al, C, N, O, P)**

| No. | Space group | SG Number | Pearson symbol | Crystal System | Prototype | Reference |
|---|---|---|---|---|---|---|
| 1 | PNNM | 58 | *oP*10 | orthorhombic | $FeB_4$ | [A1] |
| 2 | IMMM | 71 | *oI*10 | orthorhombic | $CrB_4$ | [A2] |
| 3 | C2/M | 12 | *mS*10 | monoclinic | $MnB_4$ | [A3] |
| 4 | PMMN | 59 | *oP*10 | orthorhombic | $OsB_4$ | [A4] |
| 5 | P63/MMC | 194 | *hP*20 | hexagonal | $WB_4$ | [A5] |
| 6 | P4/MBM | 127 | *tP*20 | tetragonal | $CaB_4$ | [A6] |
| 7 | PNMA | 62 | *oP*20 | orthorhombic | $MgB_4$ | [A7] |
| 8 | R-3M | 166 | *hR*15 | hexagonal | $SiB_4$ | [A8] |
| 9 | I4/MMM | 139 | *tI*10 | tetragonal | $BaAl_4$ | [A9] |
| 10 | IMM2 | 44 | *oI*30 | orthorhombic | $LaAl_4$ | [A10] |
| 11 | C1M1 | 8 | *mS*30 | monoclinic | $MoAl_4$ | [A11] |
| 12 | IMMA | 74 | *oI*20 | orthorhombic | $UAl_4$ | [A12] |
| 13 | P-1 | 2 | *aP*30 | triclinic | $MnP_4$ | [A13] |
| 14 | P-1 | 2 | *aP*15 | triclinic | $RuP_4$ | [A14] |
| 15 | PBCA | 61 | *oP*40 | orthorhombic | $ReP_4$ | [A15] |
| 16 | P-1 | 2 | *aP*10 | triclinic | $MnP_4$ | [A16] |
| 17 | C12/C1 | 15 | *mS*20 | monoclinic | $VP_4$ | [A17] |
| 18 | P41212 | 92 | *tP*20 | tetragonal | $ZnP_4$ | [A18] |
| 19 | P121/c1 | 14 | *mP*10 | monoclinic | $MgP_4$ | [A19] |
| 20 | C2221 | 20 | *oS*20 | orthorhombic | $FeP_4$ | [A20] |
| 21 | P121/c1 | 14 | *mP*30 | monoclinic | $FeP_4$ | [A21] |
| 22 | C121 | 5 | *mS*10 | monoclinic | $OsO_4$ | [A22] |
| 23 | P-43n | 218 | *cP*40 | cubic | $RuO_4$ | [A23] |

| | | | | | | |
|---|---|---|---|---|---|---|
| 24 | I12/c1 | 15 | *mS*40 | monoclinic | $VS_4$ | [A24] |
| 25 | C2/M | 12 | *mS*30 | monoclinic | $FeB_4$ | [A1] |
| 26 | P63/MMC | 194 | *hP*10 | hexagonal | $MoB_4$ | [A25] |

**References:**


[A1] A. N. Kolmogorov, Phys. Rev. Lett. 105, 217003 (2010).

[A2] A. F. Bialon, Appl. Phys. Lett. 98, 081901 (2011).

[A3] S. Andersson and J. O. Carlsson, Acta Chem. Scand. 1, 1 (1988).

[A4] M. G. Zhang, H. Y. Yan, G. T. Zhang, and H. Wang, J. Phys. Chem. C 116, 4293 (2012).

[A5] P. A. Romans and M. P. Krug, Acta Crystall. 20, 313 (1966).

[A6] R. Schmitt, B. Blaschkowski, K. Eichele, and H. J. Meyer, Inorg. Chem. 45, 3067 (2006).

[A7] A. Guette, R. Naslain, and J. Galy, Comptes Rendus Hebdomadaires des Seances de l'Academie des Sciences, Serie C, Sciences Chimiques 275, 41 (1972).

[A8] C. Brosset and B. Magnusson, Nature 187, 54 (1960).

[A9] G. Bruzzone and F. Merlo, J. Less-Common. Metals 39, 1 (1975).

[A10] I. I. Zalutskii and P. I. Kripyakevich, Dopovidi Akademii Nauk Ukrains'koi RSR, Seriya A: Fiziko-Tekhnichni ta Matematichni Nauki 4, 362 (1967).

[A11] J. A. Leake, Acta Crystall. 17, 918 (1964).

[A12] O. Tougait and H. Noel, Intermetallics 12, 219 (2004).

[A13] R. Ruehl and W. Jeitschko, Acta Crystall. B 37, 39 (1981).

[A14] D. J. Braun and W. Jeitschko, Z. Anorg. Allg. Chem. 445, 157 (1978).

[A15] W. Jeitschko and R. Ruehl, Acta Crystall. B 35, 1953 (1979).

[A16] W. Jeitschko, R. Ruehl, U. Krieger, and C. Heiden, Materials Research Bulletin 15, 1755 (1980).

[A17] W. Jeitschko, U. Floerke, and U. D. Scholz, J. Solid State Chem. 52, 320 (1984).

[A18] A. Dommann, R. E. Marsh, and F. Hulliger, J. Less-Common. Metals 152, 1 (1989).

[A19] H. G. von Schnering and G. Menge, Z. Anorg. Allg. Chem. 422, 219 (1976).

[A20] M. Sugitani, N. Kinomura, M. Koizumi, and S. Kume, J. Solid State Chem. 26, 195 (1978).

[A21] W. Jeitschko and D. J.Braun, Acta Crystall. B 34, 3196 (1978).

[A22] A. Zalkin and D. H. Templeton, Acta Crystall. 6, 106 (1953).

[A23] M. Pley and M. S. Wickleder, J. Solid State Chem. 178, 3206 (2005).

[A24] R. Allmann, I. Baumann, A.Kutoglu, H. Roesch, and E. Hellner, Naturwissenschaften 51, 263 (1964).

[A25] M. G. Zhang, H. Wang, H. B. Wang, T. Cui, and Y. M. Ma, J. Phys. Chem. C 114, 6722 (2010).

**Table S2. The nine independent elastic constants of orthorhombic transition metal tetraborides ($TmB_4$, Tm=Cr, Mn and Fe) calculated by first principles methods and compared with previous theoretical results.**

| $TmB_4$ | Reference | $C_{11}$ | $C_{22}$ | $C_{33}$ | $C_{44}$ | $C_{55}$ | $C_{66}$ | $C_{12}$ | $C_{13}$ | $C_{23}$ |
|---|---|---|---|---|---|---|---|---|---|---|
| $CrB_4$ | This study | 557 | 891 | 486 | 252 | 280 | 253 | 71 | 115 | 103 |
| | [B1] | 554 | 880 | 473 | 254 | 282 | 250 | 65 | 107 | 95 |
| | [B2] | 542 | 855 | 492 | 252 | 280 | 253 | 50 | 104 | 87 |
| | [B3] | 499 | 857 | 457 | 246 | 271 | 242 | 42 | 75 | 78 |
| $MnB_4$ | This study | 520 | 892 | 491 | 240 | 238 | 227 | 94 | 126 | 93 |
| | [B1] | 520 | 891 | 507 | 241 | 242 | 224 | 84 | 113 | 91 |
| $FeB_4$ | This study | 405 | 753 | 451 | 217 | 143 | 225 | 166 | 158 | 152 |
| | [B1] | 381 | 710 | 435 | 218 | 114 | 227 | 137 | 143 | 128 |
| | [B4] | 408 | 754 | 456 | 218 | 148 | 223 | 153 | 159 | 153 |
| | [B5] | 409 | 768 | 451 | 216 | 154 | 222 | 161 | 161 | 152 |
| | [B6] | 379 | 740 | 413 | 215 | 136 | 210 | 126 | 145 | 131 |
| | [B3] | 408 | 754 | 448 | 219 | 141 | 229 | 165 | 160 | 154 |

**References:**

[B1] H. Niu, J. Wang, X. Q. Chen, D. Li, Y. Li, P. Lazar, R. Podloucky, and A. N. Kolmogorov, Phys. Rev. B 85, 144116 (2012).

[B2] B. Li, H. Sun, C. P. Zang, and C. F. Chen, Phys. Rev. B 87, 174106 (2013).

[B3] M. Yang, Y. C. Wang, J. L. Yao, Z. P. Li, J. Zhang, L. L. Wu, H. Li, J. W. Zhang, and H. Y. Gou, J. Solid State Chem. 213, 52 (2014).

[B4] B. Li, H. Sun, and C. F. Chen, Phys. Rev. B 90, 014106 (2014).

[B5] M. Zhang, M. C. Lu, Y. H. Du, L. L. Gao, C. Lu, and H. Y. Liu, J. Chem. Phys. 140, 174505 (2014).

[B6] Y. P. Gou, Z. Fu, Y. C. Liang, Z. Zhong, and S. M. Wang, Solid St. Comm. 187, 28 (2014).

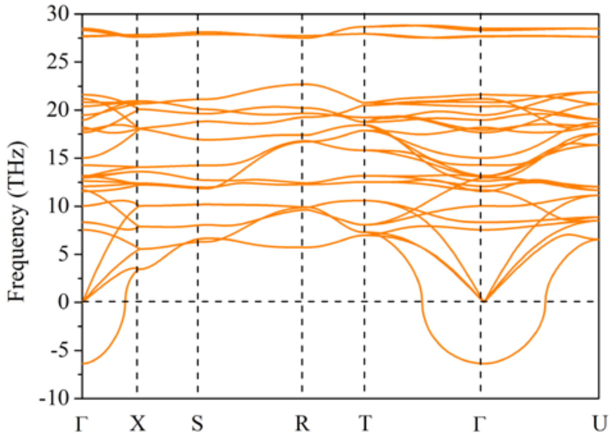


(a)

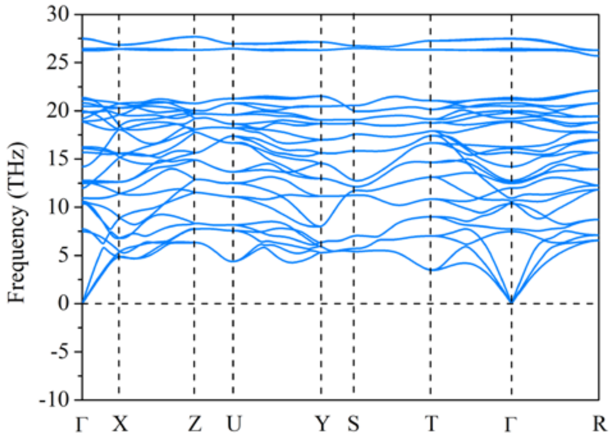


(b)

**Figure S1.** Calculated phonon dispersion curves for (a) *oI*10[71]- and (b) *oP*10[58]-$FeB_4$.

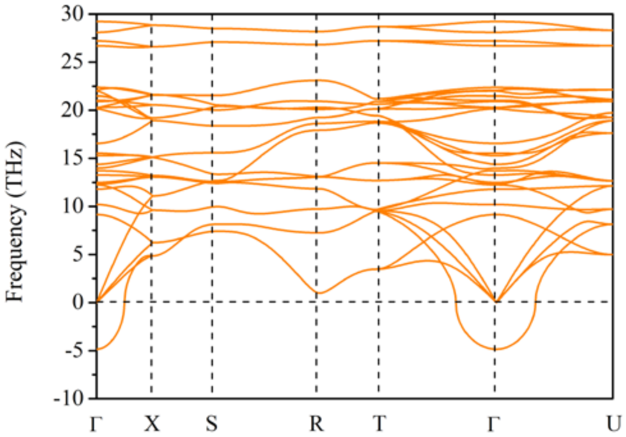


(a)

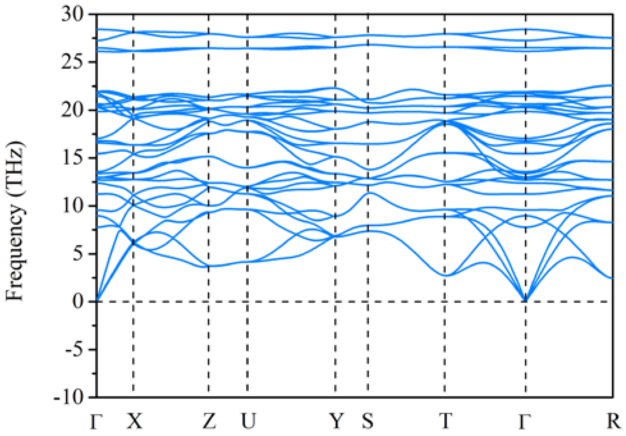


(b)

**Figure S2.** Calculated phonon dispersion curves for (a) *oI*10[71]- and (b) *oP*10[58]-$MnB_4$.

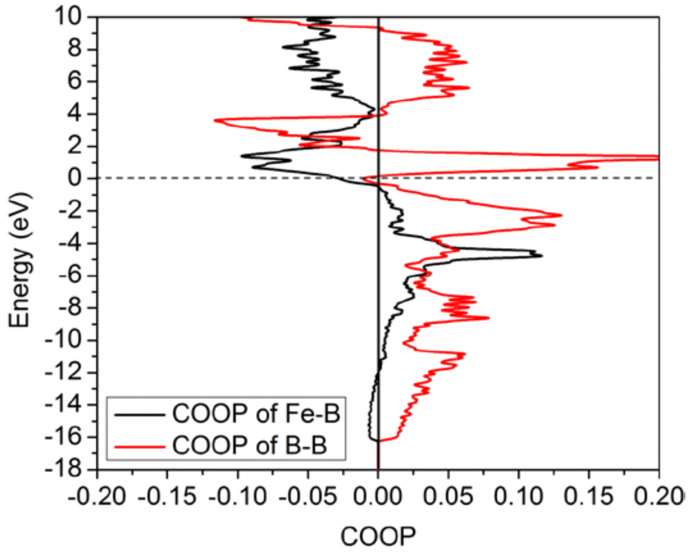


(a)

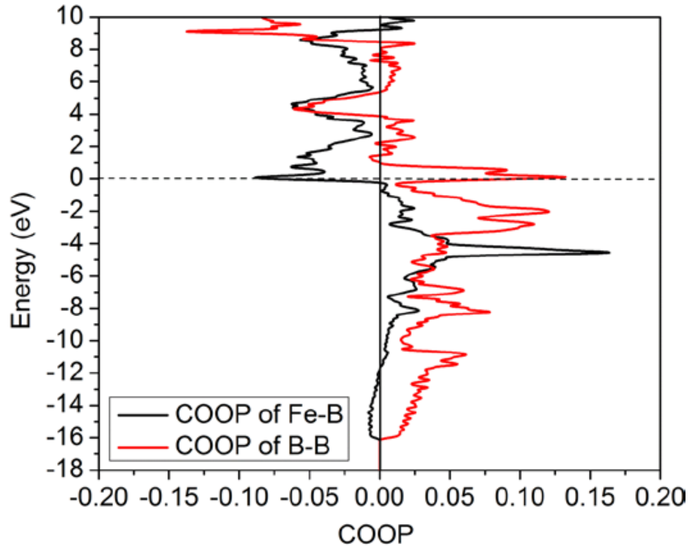


(b)

**Figure S3.** The calculated COOP curve for (a) *oI*10[71]- and (b) *oP*10[58]-$FeB_4$.

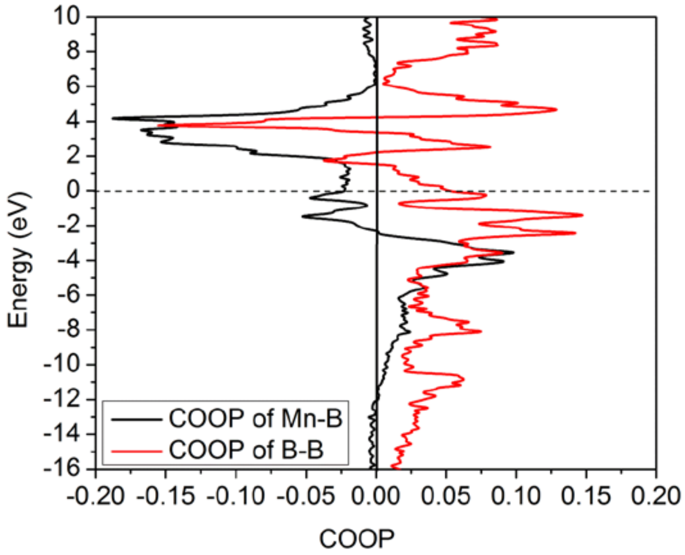


(a)

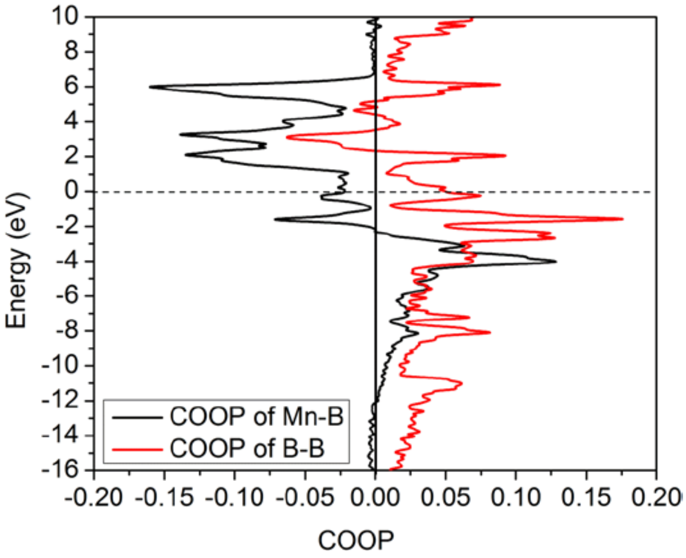


(b)

**Figure S4.** The calculated COOP curve for (a) *oI*10[71]- and (b) *oP*10[58]-$MnB_4$.

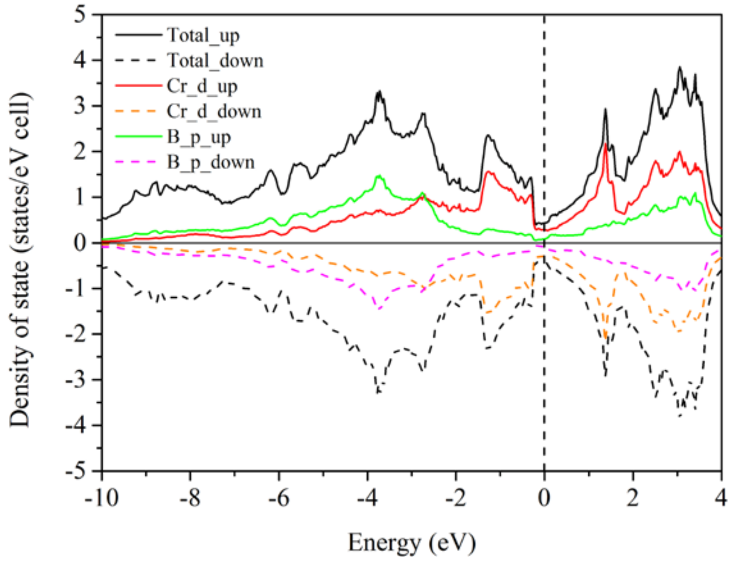


(a)

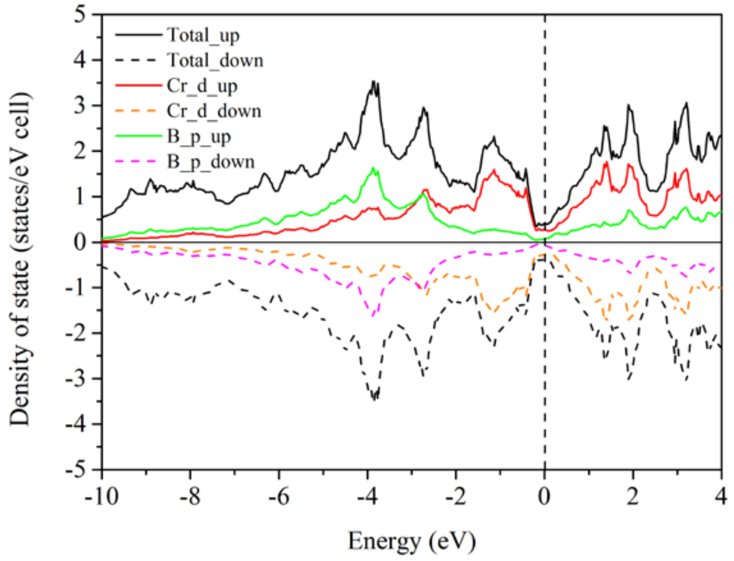


(b)

**Figure S5**. Total and partial electronic density of states of (a) *oI*10[71]-$CrB_4$, (b) *oP*10[58]-$CrB_4$. The vertical dashed lines indicate the Fermi levels.

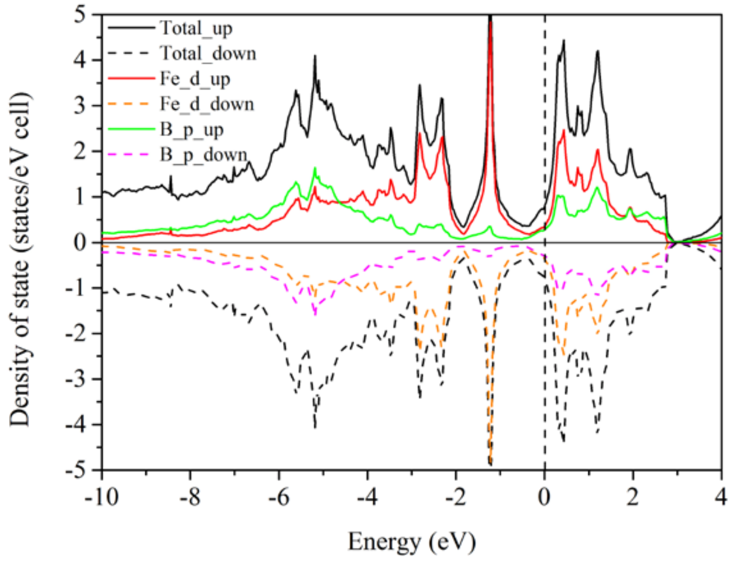


(a)

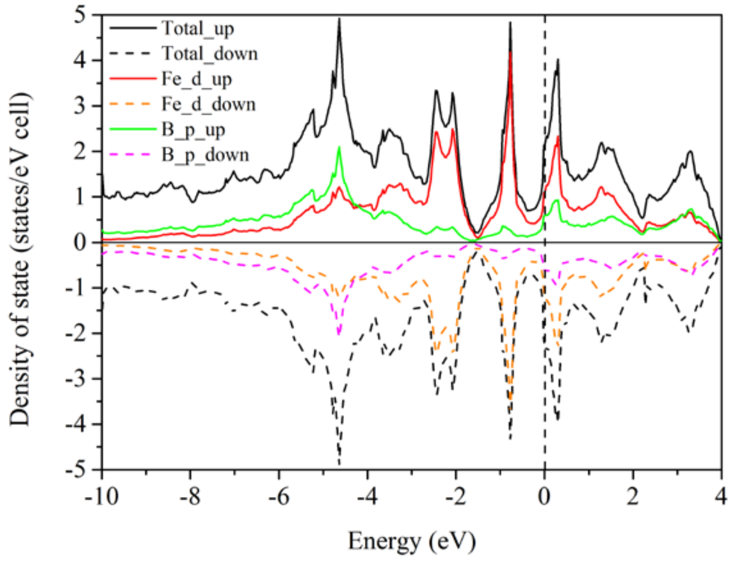


(b)

**Figure S6**. Total and partial electronic density of states of (a) *oI*10[71]-$FeB_4$ and (b) *oP*10[58]-$FeB_4$. The vertical dashed lines indicate the Fermi levels.

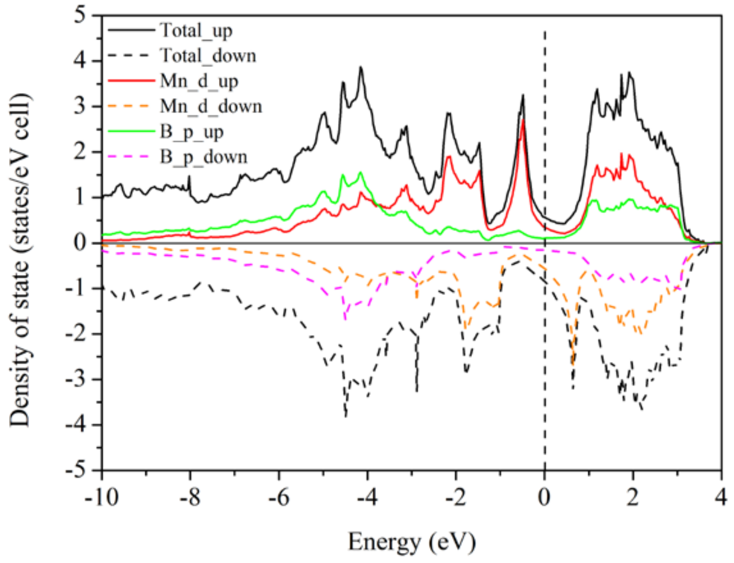


(a)

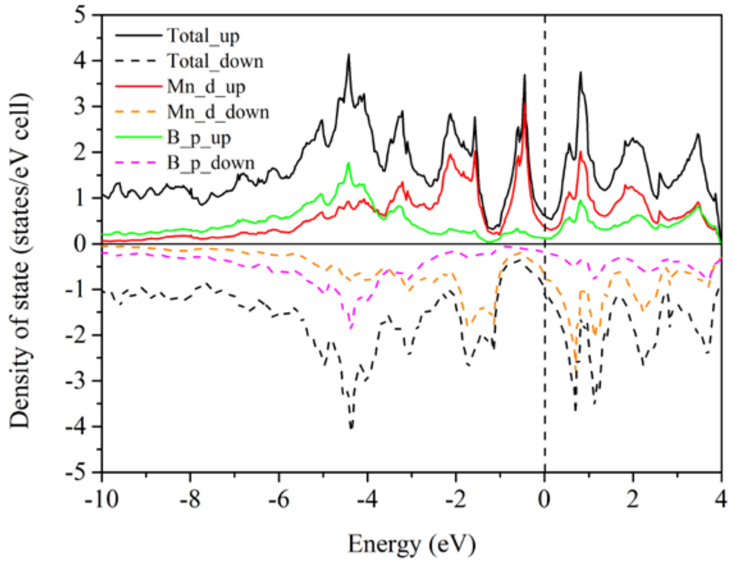


(b)

**Figure S7.** Total and partial electronic density of states of (a) *oI*10[71]-$MnB_4$ and (b) *oP*10[58]-$MnB_4$. The vertical dashed lines indicate the Fermi levels.

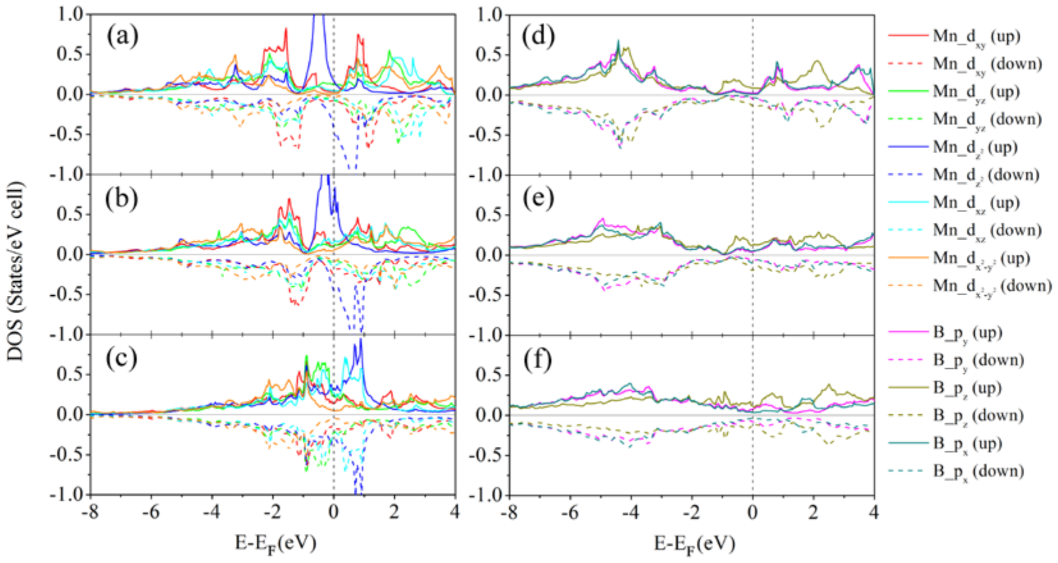


**Figure S8.** Orbital-decomposed electronic density of states of *oP*10[58]-$MnB_4$ (a) and (d) at equilibrium, (b) and (e) at a strain of 0.1951, (c) and (f) at a strain of 0.4002. The reference Cartesian coordinates is represented with x axis normal to crystallographic (1-10) plane, y axis along crystallographic [110] direction and z axis along [001] direction.
